# Predictors of Nasal Obstruction: Quantification and Assessment Using Multiple Grading Scales

**DOI:** 10.1155/2016/6945297

**Published:** 2016-05-16

**Authors:** Macario Camacho, Soroush Zaghi, Victor Certal, Jose Abdullatif, Rahul Modi, Shankar Sridhara, Anthony M. Tolisano, Edward T. Chang, Benjamin B. Cable, Robson Capasso

**Affiliations:** ^1^Otolaryngology-Head and Neck Surgery, Tripler Army Medical Center, Honolulu, HI 96859, USA; ^2^Department of Psychiatry and Behavioral Sciences, Sleep Medicine Division, Stanford Hospital and Clinics, Stanford, CA 94063, USA; ^3^Otolaryngology-Head and Neck Surgery, Division of Sleep Surgery and Medicine, Stanford Hospitals and Clinics, Stanford, CA 94304, USA; ^4^Department of Head and Neck Surgery, David Geffen School of Medicine at UCLA, Los Angeles, CA 90095, USA; ^5^Department of Otorhinolaryngology, Sleep Medicine Centre, Hospital CUF, 4100-180 Porto, Portugal; ^6^Centre for Research in Health Technologies and Information Systems (CINTESIS), University of Porto, 4200-450 Porto, Portugal; ^7^Department of Otorhinolaryngology, Hospital Bernardino Rivadavia, C1425ASQ Buenos Aires, Argentina; ^8^Department of Otolaryngology-Head and Neck Surgery, Dr. L. H. Hiranandani Hospital Mumbai, Maharashtra 400076, India; ^9^Otolaryngology-Head and Neck Surgery, Dwight D. Eisenhower Army Medical Center, Fort Gordon, GA 30905, USA

## Abstract

*Objective*. To evaluate the association between nasal obstruction and (1) demographic factors, (2) medical history, (3) physical tests, and (4) nasal exam findings.* Study Design*. Case series.* Methods*. Chart review at a tertiary medical center.* Results*. Two hundred-forty consecutive patients (52.1 ± 17.5 years old, with a Nasal Obstruction Symptom Evaluation (NOSE) score of 32.0 ± 24.1) were included. Demographic factors and inferior turbinate sizes were not associated with NOSE score or Nasal Obstruction Visual Analog Scale (NO-VAS). A significant association was found between higher NOSE score on univariate analysis and positive history of nasal trauma (*p* = 0.0136), allergic rhinitis (*p* < 0.0001), use of nasal steroids (*p* = 0.0108), higher grade of external nasal deformity (*p* = 0.0149), higher internal nasal septal deviation grade (*p* = 0.0024), and narrow internal nasal valve angle (*p* < 0.0001). Multivariate analysis identified the following as independent predictors of high NOSE score: NO-VAS: ≥50 (Odds Ratio (OR) = 17.6 (95% CI 5.83–61.6), *p* < 0.0001), external nasal deformity: grades 2–4 (OR = 4.63 (95% CI 1.14–19.9), *p* = 0.0339), and allergic rhinitis: yes (OR = 5.5 (95% CI 1.77–18.7), *p* = 0.0041).* Conclusion*. Allergic rhinitis, NO-VAS score ≥ 50, and external nasal deformity (grades 2–4) were statistically significant independent predictors of high NOSE scores on multivariate analysis. Inferior turbinate size was not associated with NOSE scores or NO-VAS.

## 1. Introduction

Nasal obstruction is a frequent complaint, which affects breathing during wakefulness and sleep [1]. Systematic evaluation of nasal obstruction remains challenging due to the high number of variables and factors that can contribute to nasal obstruction. These can be grouped into four major categories: (1) demographic factors, (2) medical history, (3) physical tests, and (4) nasal exam findings. Notably, nasal exam findings do not always correlate with patient symptoms. For example, some patients with internal nasal septal deviations, narrow internal nasal valve angles, and/or large inferior turbinates may have no or few complaints of nasal obstruction, while other patients may complain of nasal obstruction despite the presence of minimal objective anatomical abnormalities. These observations are well known to otolaryngologists, but the efforts to quantify obstruction in a way that allows for systematic study have been a long term challenge. Several grading scales and classification systems (for nasal physical exam findings) and questionnaires (for nasal obstruction) have been developed over the years to assist in the quantification and assessment of nasal obstruction.

The Nasal Obstruction Symptom Evaluation (NOSE) scale [3] developed by Stewart et al. is a validated quality of life instrument which quantifies nasal obstruction and is commonly used in the international literature. The NOSE scale questionnaire is composed of five questions. Each question is graded on a Likert scale from 0 (not a problem) to 4 (severe problem), and the final summed score is multiplied by 5 so that the total score ranges from 0 to 100 (0 = no obstruction, 100 = severe obstruction) [3]. Additionally, the Nasal Obstruction Visual Analog Scale (NO-VAS) is another reliable tool to quantify nasal obstruction in the absence of rhinomanometry and has a very strong direct relationship with nasal airflow resistance [4]. NO-VAS is generally performed by having patients quantify their perceived nasal obstruction using a continuous scale from 0 to 10 in which 0 corresponds to no obstruction and 10 corresponds to complete obstruction [4]. Additionally, the nasal anatomy can be evaluated by using grading scales, such as the inferior turbinate classification system, in which there are 4 grades that correspond to the space occupied by the anterior aspect of the inferior turbinate relative to the total airway space at that location [2].

The objective of this study was to evaluate the association of demographic factors, medical history, physical tests, and nasal exam findings with nasal obstruction using the NOSE score and the NO-VAS.

## 2. Materials and Methods

The Stanford University Institutional Review Board provided written approval for the protocol. This study is a retrospective case series of 240 consecutive patients evaluated in the Stanford Sleep Clinic between February 1st and June 30th, 2014, by a single board certified otolaryngologist (M.C.) specializing in sleep surgery and sleep medicine. History and physical examination data were cataloged using Microsoft® Excel® 2013 (Redmond, WA, USA). JMP 11.2 Pro (SAS Institute Inc., Cary, NC) was used for statistical analysis. The age, gender, body mass index (BMI), and ethnicity of the patients were recorded. The following items were assessed on a yes or no scale for medical history: history of nasal trauma, prior nasal surgery, history of allergic rhinitis, use of nasal steroids, use of nasal antihistamines, and use of oral antihistamines.

**Figure 1:**
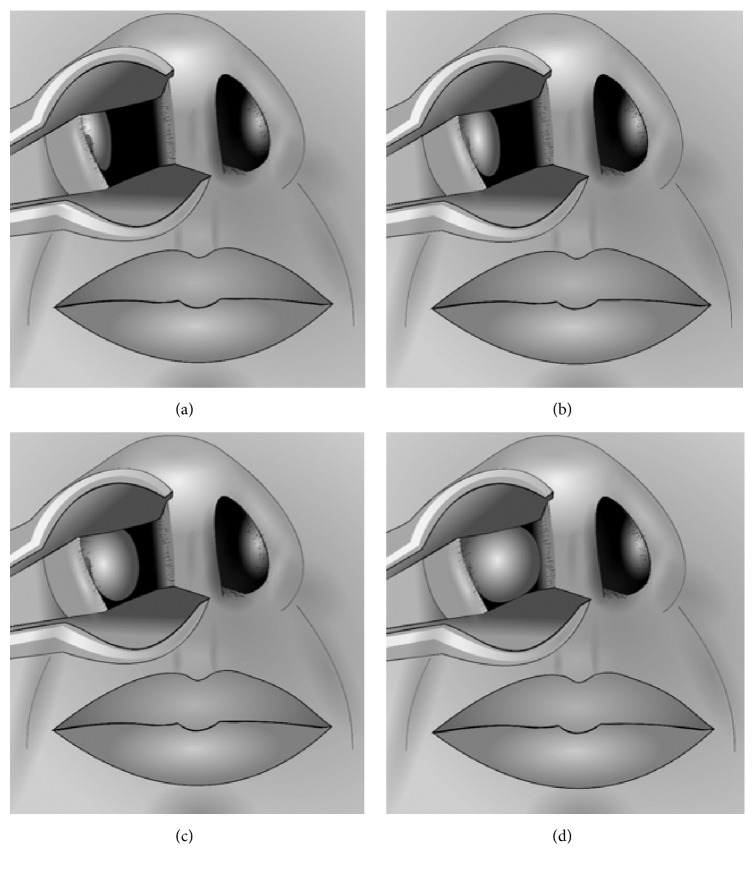
Inferior turbinate sizes. (a) Grade 1 (0%–25% of total airway space). (b) Grade 2 (26%–50% of total airway space). (c) Grade 3 (51%–75% of total airway space). (d) Grade 4 (76%–100% of total airway space). Reproduced with permission [2].

A detailed physical examination of the nasal passages was performed via anterior rhinoscopy using a simple handheld otoscope without distorting the patients' anatomy. Assessment was performed of external nasal deformity, internal nasal septal deviation, internal nasal valve angle, internal nasal valve collapse, and inferior turbinate size using ordinal scales ranging from 1 to 4. Inferior turbinate size was based on the degree of obstruction caused by the anterior aspect of the inferior turbinate relative to the total airway space and was graded as 0–25%, 26–50%, 51–75%, and 76–100%; see Figure 1 [2]. External nasal deformity was graded as none, mild, moderate, and severe. Internal nasal septal deviation was graded as 0–25% deflection, 26–50% deflection, 51–75% deflection, and 76–100% deflection (based on deflection from midline toward the lateral wall). Internal nasal valve angle was graded as <5 degrees, 5 to <10 degrees, 10 to <15 degrees, and 15 or more degrees. Internal nasal valve collapse was graded as no collapse, mild collapse (<33%), moderate collapse (33–66%), and severe collapse (>66%) [5].

Additionally, patients were asked to rate the degree of nasal obstruction at the time of the physical exam using a modified Nasal Obstruction Visual Analog Scale (NO-VAS) from 0 to 10 in which 0 corresponds to no obstruction and 10 corresponds to complete obstruction (converted to 0 to 100% obstruction) in each of three conditions: both nostrils open, left nostril open (cover right), and right nostril open (cover left) [4]. The Cottle sign (Cottle maneuver) was performed to assess the subjective effect on nasal airflow and graded 1–4 as no improvement, mild improvement, moderate improvement, and significant improvement [6]. The external nasal deformities, cephalocaudal internal nasal septal deviations, and anteroposterior internal nasal septal deviations were classified as C-shaped, reverse C-shaped, S-shaped, or reverse S-shaped if a deviation was present [7].

Distribution of patients' characteristics, medical history, and nasal exam findings are reported using the percent total for nominal and ordinal data and mean ± standard deviation (M ± SD) for continuous data. Univariate analysis was performed to assess an association with the NOSE score [3] using Pearson correlation for continuous variables, ANOVA for multinomial and ordinal data, and Student's *t*-test for binomial data. Multivariate analysis was performed with a nominal logistic model to include each of the variables found to have a significant association on univariate analysis: NO-VAS score, internal nasal valve angle, external nasal deformity, history of allergic rhinitis, effect of the Cottle maneuver, positive nasal septal deviation, and history of nasal trauma. Continuous and ordinal data were transformed into binomial data using cut-offs and were guided by using the Connecting Letters Report of ANOVA, Compare Means, Each Pair function of JMP. Statistical significance was defined as a *p* value < 0.05.

## 3. Results

There were 240 patients included in this study. The M ± SD for age was 52.1 ± 17.5 years and for BMI was 29.0 ± 6.8 kg/m^2^. There were 159 males (66.3%) and 81 females (33.7%). See Table 1 for summary of patient demographic characteristics. The inferior turbinate sizes were averaged for all 240 patients (480 inferior turbinates) and the M ± SD were 2.37 ± 1.03. These were subcategorized by race, Asian: 2.78 ± 0.81 (*n* = 94 turbinates), Black: 3.00 ± 0.66 (*n* = 20 turbinates), Caucasian: 2.19 ± 0.89 (*n* = 332 turbinates), and Latino: 2.53 ± 0.97 (*n* = 34 turbinates).

The mean NOSE score for this population was 32.0 ± 24.1 (range: 0/100 to 92.5/100) corresponding to overall mild to moderate complaints of symptomatic nasal obstruction. None of the demographic factors were found to have a significant association with NOSE scores. For medical history, a positive history of nasal trauma (*p* = 0.0136), allergic rhinitis (*p* < 0.0001), and use of nasal steroids (*p* = 0.0108) were significantly associated with higher NOSE scores on univariate analysis. The following nasal physical exam findings were also associated with higher NOSE scores on univariate analysis: higher grade of external nasal deformity (Odds Ratio (OR) = 3.59, *p* = 0.0002), higher grade of internal nasal septal deviation (OR = 2.05, *p* = 0.0168), and narrow internal nasal valve angle (OR = 4.34, *p* < 0.0001). In addition, the clinical test findings associated with high NOSE scores were NO-VAS patient subjective sensation of nasal obstruction (OR = 11.1, *p* < 0.0001) and significant improvement with the Cottle maneuver on subjective sensation of nasal airflow (OR = 2.28 (95% CI = 1.03), *p* = 0.0399). There was no significant relationship between the classification of external nasal deformity and internal septal deviation on NOSE scores. See Tables 2(a) and 2(b).

Multivariate analysis was used to develop a nominal logistic model with seven variables in which three clinical factors were identified as statistically significant independent predictors of high NOSE scores: NO-VAS: ≥50 (OR = 17.6 (95% CI 5.83–61.6), *p* < 0.0001), external nasal deformity: grades 2–4 (OR = 4.63 (95% CI 1.14–19.9), *p* = 0.0339), and allergic rhinitis: yes (OR = 5.5 (95% CI 1.77–18.7), *p* = 0.0041); see Table 3. Exploratory analysis with backward elimination revealed that the variable “internal nasal valve angle” was significant on multivariate analysis only when external nasal deformity was excluded, suggesting that the two variables overlap to a significant degree. Pearson chi square analysis demonstrated a significant association between external nasal deformity (grades 2–4) and internal nasal valve angle < 10 degrees (either <5 degrees or 5 to <10 degrees) (OR = 3.33 (95% CI 1.67–6.64), *p* = 0.0004). Univariate analysis with one-way ANOVA showed that external nasal deformity and internal nasal valve angles were significantly associated with NO-VAS scores; see Table 4. Inferior turbinate size was not associated with NOSE scores or any of the NO-VAS measures.

## 4. Discussion

There are four main findings in this study. First, physical exam tests were significantly associated with nasal obstruction. This study demonstrated that the presence of an external nasal deformity and a narrow internal nasal valve angle are associated with higher NO-VAS scores. In some cases, especially when nasal steroids do not improve nasal breathing, a referral to an otolaryngologist may be warranted, as some patients may have fixed anatomical obstructions which could be improved with surgery. Examples include a narrow internal nasal valve angle and/or internal nasal septal deviation. We found that 89% of patients reported at least mild improvement in nasal breathing and nearly half of all patients reported moderate or significant improvement with the Cottle maneuver. For patients with no improvement in breathing with the Cottle maneuver, the NOSE score was very low (15.0 ± 17.9), while those with mild (30.3 ± 22.9), moderate (37.4 ± 24.1), or significant (37.4 ± 27.2) improvement with the Cottle maneuver had higher NOSE scores. Given that the Cottle maneuver improved the subjective sensation of nasal airflow in 89% of patients in this study, this test may not be as helpful in determining the site of nasal obstruction, especially with regard to trying to determine if a specific nasal surgery would benefit the patient. However, it potentially could assist with determining who might benefit from surgery, generally.

Second, there are several different anatomical variables that may contribute to nasal obstruction. By using grading scales, this study was able to determine the specific grades of nasal anatomical variables that were associated with nasal obstruction. For example, we demonstrated that internal nasal septal deviations contribute significantly to nasal obstruction. More importantly, we identified a “severity-dependent” relationship, such that the average NOSE score increased with higher grade deflections. In contrast, the shape of the internal nasal septal deviations (in either the cephalocaudal or anteroposterior dimensions) was not associated with nasal obstruction, demonstrating that the severity of the septal deviation is most important. Furthermore, patients with an external nasal deformity were found to be highly likely to also have narrowing of the internal nasal valve, and narrow angles were associated with higher NOSE scores in a similar severity-dependent relationship. This underscores the importance of examination of the internal nasal valve angle during evaluation of the upper airway, particularly if an external nasal deformity is present.

Third, although the inferior turbinates seemingly contribute significantly to the overall nasal cavity airway space at the level of the internal nasal valve, the size of the inferior turbinates was not associated with either the NOSE score or NO-VAS measures. This study demonstrated that inferior turbinates are generally of larger sizes for Asians (2.78 ± 0.81) and Blacks (3.00 ± 0.66), while they tend to be smaller in Caucasians (2.19 ± 0.89) and are in between for Latinos (2.53 ± 0.97). Caution, therefore, should always be exercised in evaluating a patient with isolated turbinate hypertrophy, particularly if they do not have an elevated NOSE or NO-VAS score. Moreover, additional anatomic contributors to nasal obstruction should be sought in the patient with presumed isolated turbinate hypertrophy as the sole cause for nasal obstruction. In a systematic review, Rhee et al. identified several studies reporting a significant decrease in the NOSE score after inferior turbinoplasties were performed [8]. Therefore, inferior turbinoplasties alone may benefit patients with nasal obstruction and the isolated nasal exam finding of inferior turbinate hypertrophy. Inferior turbinoplasties are commonly performed at the time of septoplasties, septorhinoplasties, or sinus surgeries in order to increase the size of the nasal airway, which provides the additional benefit of increasing continuous positive airway pressure (CPAP) device use and decreasing therapeutic CPAP treatment pressures [9].

Lastly, we would encourage the use of questionnaires, grading scales, and classification systems as a means to help identify specific factors (demographics, medical history, physical tests, and nasal exam findings) that contribute to nasal obstruction. The use of these tools allows for the treatment (medical or surgical) to be evaluated in a systematic fashion before and after the intervention. The use of grading scales and the reporting of outcomes (with means and standard deviations) based on grades can also facilitate future research to include meta-analyses. Currently, there are several grading scales and classification systems in the published literature. In the head and neck, it is common to use four grades per subsite and this promotes high intra- and interrater reliability during validation testing [2]. Some head and neck subsites such as tonsil sizes [10] are commonly incorporated into the medical record. In this study, we referenced questionnaires and nasal exam classification systems based on a 1 to 4 grading scale, which have easily been incorporated into the standard physical examination. Future research could be aimed at evaluating the general population (especially in patients with no complaints of nasal obstruction) in order to help establish normative data.

## 5. Limitations

This study was a retrospective review, and, like any retrospective review, the authors are limited to what has been documented previously. However, because the first author incorporated a detailed upper airway exam to include the use of grading scales for nasal examinations, these were consistently documented into the medical record in a standardized way. The findings from this study are based on a single institution; the goal of the authors is to perform future multi-institutional studies evaluating the effect of multiple variables on nasal obstruction.

## 6. Conclusion

Allergic rhinitis, NO-VAS score ≥ 50, and external nasal deformity were statistically significant independent predictors of high NOSE scores on multivariate analysis. Inferior turbinate size was not associated with NOSE scores or NO-VAS.

## Figures and Tables

**Table 1:** Summary of patient characteristics and association with NOSE score.

	Percent total or mean ± SD (*n* = number of patients)	NOSE score mean ± SD or Pearson's *R*	*p* value, statistical test
			‡ (one-way ANOVA)
			† (Student's *t*-test)
			¶ (Pearson correlation)
			*∗* = statistical significance
*Demographics *			
Age (years)	52.1 ± 17.5 years (*n* = 240)	*R* ^2^ = 8.5 × 10^−5^ No or negligible relationship	*p* = 0.8843, ¶
Gender (%)			
Male	66.3% (*n* = 159)	30.4 ± 22.5	*p* = 0.1486, †
Female	33.7% (*n* = 81)	35.2 ± 26.8	
BMI (kg/m^2^)	29.0 ± 6.8 kg/m^2^ (*n* = 240)	*R* ^2^ = 0.0007 No or negligible relationship	*p* = 0.6815, ¶
Ethnicity (%)			
Caucasian	69.1% (*n* = 166)	33.4 ± 24.5	*p* = 0.2572, ‡
Asian	12.5% (*n* = 30)	26.8 ± 23.5	
Hispanic	7.1% (*n* = 17)	25.2 ± 20.3	
Indian	6.3% (*n* = 15)	27.1 ± 19.0	
Black	4.1% (*n* = 10)	44.3 ± 29.9	
Pacific Islander	0.83% (*n* = 2)	27.5 ± 3.5	

*Medical history*			
History of nasal trauma (%)			
Yes	18.7% (*n* = 45)	**40.0 ± 23.6**	**p** ** = 0.0136**, †*∗*
No	81.3% (*n* = 195)	**30.2 ± 23.9**	
Prior nasal surgery (%)			
Yes	22.5% (*n* = 54)	36.3 ± 23.6	*p* = 0.1421, †
No	77.5% (*n* = 186)	30.8 ± 24.2	
Allergic rhinitis (%)			
Yes	33.8% (*n* = 81)	**41.2 ± 25.8**	**p** ** < 0.0001**, †*∗*
No	66.3% (*n* = 159)	**27.3 ± 21.8**	
Nasal steroids (%)			
Yes	14.6% (*n* = 35)	**41.5 ± 24.9**	**p** ** = 0.0108**, †*∗*
No	85.4% (*n* = 205)	**30.4 ± 23.7**	
Nasal antihistamines (%)			
Yes	10.8% (*n* = 26)	38.0 ± 25.0	*p* = 0.1820, †
No	89.1% (*n* = 214)	31.3 ± 23.9	
Oral antihistamines (%)			
Yes	5.0% (*n* = 12)	31.5 ± 23.8	*p* = 0.1379, †
No	95.0% (*n* = 228)	42.0 ± 28.8	

**(a):** 

	Percent total (*n* = number of patients)		NOSE score by subgroup (mean ± SD)		*p* value	
					(one-way ANOVA)	
					*∗* = statistical significance	
*External nasal deformity*						
Grade 1: none	82.5% (*n* = 198)		30.1 ± 22.8		**p** ** = 0.0149** ^*∗*^	
Grade 2: mild	9.2% (*n* = 22)		34.5 ± 25.3			
Grade 3: moderate	7.9% (*n* = 19)		48.5 ± 30.2			
Grade 4: severe	0.4% (*n* = 1)		35.0			

*Nasal septum deviation*						
Grade 1: 0 to 25% deflection	53.3% (*n* = 128)		27.7 ± 22.0		**p** ** = 0.0024** ^*∗*^	
Grade 2: 26–50% deflection	31.6% (*n* = 76)		33.5 ± 22.9			
Grade 3: 51–75% deflection	8.3% (*n* = 20)		43.3 ± 32.8			
Grade 4: 76–100% deflection	6.7% (*n* = 16)		45.9 ± 25.4			

*Internal nasal valve angle*	*Right*	*Left*	*Right*	*Left*	*Right*	*Left*
Grade 1: <5 degrees	3.8% (*n* = 9)	4.2% (*n* = 10)	66.1 ± 18.3	58.0 ± 27.4	**p** ** < 0.0001**	**p** ** < 0.0001**
Grade 2: 5 to <10 degrees	18.1% (*n* = 43)	18.1% (*n* = 43)	44.3 ± 25.5	44.1 ± 24.8		
Grade 3: 10 to <15 degrees	51.9% (*n* = 123)	53.6% (*n* = 127)	29.1 ± 21.9	28.9 ± 21.5		
Grade 4: 15 or more degrees	26.2% (*n* = 62)	24.1% (*n* = 57)	24.8 ± 21.6	25.7 ± 22.6		

*Internal nasal valve collapse*	*Right*	*Left*	*Right*	*Left*	*Right*	*Left*
Grade 1: no collapse	74.1% (*n* = 178)	74.2% (*n* = 178)	No significant difference between groups		*p* = 0.4210	*p* = 0.1053
Grade 2: mild collapse ≤ 33%	17.9% (*n* = 43)	18.3% (*n* = 44)				
Grade 3: moderate collapse = 34–66%	7.5% (*n* = 18)	6.7% (*n* = 16)				
Grade 4: severe collapse ≥ 67%	0.4% (*n* = 1)	8.3% (*n* = 2)				

*Inferior turbinate size*	*Right*	*Left*	*Right*	*Left*	*Right*	*Left*
Grade 1: 0–25% AP nasal airway space	27.9% (*n* = 67)	25.0% (*n* = 60)	No significant difference between groups		*p* = 0.9472	*p* = 0.1618
Grade 2: 26–50% AP nasal airway space	24.1% (*n* = 58)	27.1% (*n* = 65)				
Grade 3: 51–75% AP nasal airway space	30.0% (*n* = 72)	32.9% (*n* = 79)				
Grade 4: 76–100% AP nasal airway space	17.9% (*n* = 43)	15.0% (*n* = 36)				

**(b):** 

	Subgroup % total or mean ± SD (*n* = number)	NOSE score mean ± SD or Pearson's *R*	*p* value, statistical test
			‡ (one-way ANOVA)
			¶ (Pearson correlation)
			*∗* = statistical significance
*Nasal obstruction visual analog scale (NO-VAS): 0–100*			
Bilateral (both nostrils open)	22.3 ± 23.8	*R* ^2^ = 0.38; moderate positive relationship	*p* < 0.0001, ¶*∗*
Left nostril (cover right)	27.6 ± 28.0	*R* ^2^ = 0.37; moderate positive relationship	*p* < 0.0001, ¶*∗*
Right nostril (cover left)	23.0 ± 25.7	*R* ^2^ = 0.21; weak positive relationship	*p* < 0.0001, ¶*∗*

*Cottle maneuver effect on nasal airflow*			
Grade 1: no improvement	11.1% (*n* = 16)	15.0 ± 17.9	*p* = 0.0087, ‡*∗*
Grade 2: mild improvement	39.6% (*n* = 57)	30.3 ± 22.9	
Grade 3: moderate improvement	25.7% (*n* = 37)	37.4 ± 24.1	
Grade 4: significant improvement	23.6% (*n* = 34)	37.4 ± 27.2	

*Classification of external nasal deformities*			
C-shaped	26.7% (*n* = 4)	No significant difference between groups	*p* = 0.8352, ‡
Reverse C-shaped	60.0% (*n* = 9)		
S-shaped	13.3% (*n* = 2)		
Reverse S-shaped	0% (*n* = 0)		

*Classification of septal deviations: cephalocaudal dimension*			
C-shaped	51.4% (*n* = 35)	No significant difference between groups	*p* = 0.5270, ‡
Reverse C-shaped	36.8% (*n* = 25)		
S-shaped	7.4% (*n* = 5)		
Reverse S-shaped	4.4% (*n* = 3)		

*Classification of septal deviations: anteroposterior dimension*			
C-shaped	50.0% (*n* = 32)	No significant difference between groups	*p* = 0.6841, ‡
Reverse C-shaped	36.0% (*n* = 23)		
S-shaped	10.9% (*n* = 7)		
Reverse S-shaped	3.1% (*n* = 2)		

**Table 3:** Clinical factors related to high NOSE score (≥50, “moderate to severe problem”): results of univariate and multivariate analysis.

Prognostic factor	Univariate analysis			Multivariate analysis		
	Odds ratio	95% confidence interval	*p* value (Pearson's chi square)	Odds ratio	95% confidence interval	*p* value (Pearson's chi square)
Nasal obstruction visual analog scale: ≥50	11.1	4.40–28.11	*p* < 0.0001^*∗*^	17.6	5.83–61.6	*p* < 0.0001^*∗*^
Internal nasal valve angle: <10 degrees (grade 1 or 2)^‡^	4.34	2.31–8.12	*p* < 0.0001^*∗*^	NS	NS	*p* = 0.2433
External nasal deformity: mild to severe (grades 2–4)	3.59	1.79–7.22	*p* = 0.0002^*∗*^	4.63	1.14–19.9	*p* = 0.0339^*∗*^
Allergic rhinitis: yes	3.36	1.83–6.16	*p* < 0.0001^*∗*^	5.5	1.77–18.7	*p* = 0.0041^*∗*^
Use of nasal steroids: yes	2.30	1.08–4.89	*p* = 0.0266^*∗*^	NS	NS	*p* = 0.2262
Cottle maneuver: moderate to significant improvement (grade 3 or 4)	2.28	1.03–5.07	*p* = 0.0399^*∗*^	NS	NS	*p* = 0.2862
Nasal septal deviation: mild to severe (grades 2–4)	2.05	1.13–3.72	*p* = 0.0168^*∗*^	NS	NS	*p* = 0.1906
History of nasal trauma: yes	1.89	0.94–3.80	*p* = 0.0697	NS	NS	*p* = 0.6106

^‡^At least one nasal valve (right or left) with angle < 10 degrees.

^*∗*^Statistical significance.

**Table 4:** Association of nasal physical exam findings with NO-VAS scores at time of exam.

	Percent total (*n* = number of patients)		NO-VAS score by subgroup (mean ± SD)		*p* value (one-way ANOVA) *∗* = statistical significance	
*External nasal deformity*			*NO-VAS: bilateral*			
Grade 1: none	88.6% (*n* = 132)		20.6 ± 23.9		**p** ** = 0.0099** ^*∗*^	
Grade 2: mild	0%		N/A			
Grade 3: moderate	11.4% (*n* = 17)		36.3 ± 18.2			
Grade 4: severe	0%		N/A			

*Nasal septum deviation*						
Grade 1: 0 to 25% deflection	60.4% (*n* = 90)		No significant difference between groups		*p* = 0.0612	
Grade 2: 26–50% deflection	21.4% (*n* = 32)					
Grade 3: 51–75% deflection	12.1% (*n* = 18)					
Grade 4: 76–100% deflection	6.0% (*n* = 9)					

*Internal nasal valve angle*	*Right*	*Left*	*NO-VAS: right*	*NO-VAS: left*	*Right*	*Left*
Grade 1: <5 degrees	4.1% (*n* = 6)	6.1% (*n* = 9)	54.8 ± 29.9	48.2 ± 34.2	**p** ** = 0.0023** ^*∗*^	**p** ** = 0.0014** ^*∗*^
Grade 2: 5 to <10 degrees	17.8% (*n* = 26)	17.8% (*n* = 26)	24.9 ± 20.1	39.7 ± 24.5		
Grade 3: 10 to <15 degrees	43.8% (*n* = 64)	45.8% (*n* = 67)	23.8 ± 26.1	25.6 ± 26.1		
Grade 4: 15 or more degrees	34.2% (*n* = 50)	30.1% (*n* = 44)	15.6 ± 23.0	18.3 ± 27.2		

*Internal nasal valve collapse*	*Right*	*Left*	*Right*	*Left*	*Right*	*Left*
Grade 1: no collapse	86.5% (*n* = 128)	86.6% (*n* = 129)	No significant difference between groups		*p* = 0.6166	*p* = 0.2666
Grade 2: mild collapse ≤ 33%	4.0% (*n* = 6)	3.4% (*n* = 5)				
Grade 3: moderate collapse = 34–66%	10.0% (*n* = 15)	9.4% (*n* = 14)				
Grade 4: severe collapse ≥ 67%	0%	0.7% (*n* = 1)				

*Inferior turbinate size*	*Right*	*Left*	*Right*	*Left*	*Right*	*Left*
Grade 1: 0–25% AP nasal airway space	26.8% (*n* = 40)	22.1% (*n* = 33)	No significant difference between groups		*p* = 0.1487	*p* = 0.9494
Grade 2: 26–50% AP nasal airway space	24.8% (*n* = 37)	22.8% (*n* = 34)				
Grade 3: 51–75% AP nasal airway space	30.2% (*n* = 45)	40.9% (*n* = 61)				
Grade 4: 76–100% AP nasal airway space	18.1% (*n* = 27)	14.0% (*n* = 21)				
